# The Relationship of Technoference in Conjugal Interactions and Child Smartphone Dependence: The Chain Mediation between Marital Conflict and Coparenting

**DOI:** 10.3390/ijerph191710949

**Published:** 2022-09-02

**Authors:** Tingting Shao, Chengwei Zhu, Xi Quan, Haitao Wang, Cai Zhang

**Affiliations:** 1 Institute of Early Childhood Education, Faculty of Education, Beijing Normal University, Beijing 100875, China; 2 Collaborative Innovation Center of Assessment for Basic Education Quality, Beijing Normal University, Beijing 100875, China; 3 Department of Education, Ocean University of China, Qingdao 266100, China

**Keywords:** technoference in conjugal interactions, marital conflict, coparenting, child smartphone dependence, chain mediation

## Abstract

With the increasing use of smartphones in our lives, technoference has become a new threat to family relationships and child development. The present study explored the impact of technoference in conjugal interactions on child smartphone dependence and its underlying mechanism. The participants were 6923 fourth grade children (55.0% boys; the average age was 10.60 years) in 545 primary schools and their parents (35.3% fathers). We found that technoference in conjugal interactions was significantly associated with child smartphone dependence. Technoference in conjugal interactions indirectly affected child smartphone dependence through marital conflict or coparenting and a chain mediation between marital conflict and coparenting. These findings support the spillover theory and provide relevant empirical evidence and advance our understanding of parental functioning on child smartphone dependence within the Chinese culture.

## 1. Introduction

With the development of information technology, digital technology brings convenience to our lives and causes various practical technological interference problems. Smartphones are considered the devices with the highest frequency of interference [1]. The technical interference caused by smartphone use in the home has become a new threat to family relationships and child development [2]. Technical interference, also known as technoference, refers to the phenomenon of interruptions in human interactions caused by the interference of electronic devices, such as televisions, mobile phones, and computers [3]. Technoference in this study specifically refers to the interruption or interference of couples’ interactions perceived by parents due to their spouse’s use of smartphones.

Previous studies on family technoference mostly focused on the technological interference caused by parents’ mobile phone use during parent–child interactions. Studies have shown that technoference caused by parents may increase their children’s risk of smartphone dependence through a variety of pathways [4], including bad habits that demonstrate device use [5], family conflict [4], a decrease in parent–child interactions [5], and showing hostility toward the need for children to seek attention [6]. Recent studies of children’s screen activities during parent–child interactions have found that the use of screen devices has become a standard practice in modern parenting; sometimes screen devices can be a supportive force for parenting, but most of the time inappropriate use of screen devices can have an impact on the quality of parenting [7,8]. With the increasing use of smartphones and other technological devices in families, will technoference in couples’ interactions affect the formation of children’s smartphone dependence, and what are its mechanisms? These are issues that have been overlooked before but are worth exploring.

According to the expectancy violation communication theory [9], individuals have expectations of others’ behaviors during interpersonal interactions, and if an individual perceives that someone is violating those expectations, this can prompt negative reactions. Indeed, studies has shown that perceived partner phubbing or technoference can lead to negative effects [10], particularly the disruption of couples’ relationships and parenting quality. At the same time, according to the spillover effect, the influence of the destruction of the relationship between husband and wife in the couple subsystem will spill over to the parent subsystem and affect the parental coparenting behavior. The harmonious relationship between husband and wife and effective parenting are important supports for children from the family, which are closely related to the development of children’s problem behaviors [11]. The theory of compensatory advantage indicates that offline psychological needs can be met through online networks. When an individual’s basic psychological needs cannot be met, it is often accompanied by more negative coping styles. For example, children who cannot meet their psychological needs in the family will turn to the Internet to seek compensation and even develop smartphone-dependence behavior [12]. In fact, if children cannot get care and warmth from the family environment, especially the interaction and nurturing behaviors of parents, then children will seek psychological satisfaction from other places, which leads to the occurrence of smartphone-dependence behavior.

Children are particularly dependent on their parents, especially their parents’ problem behavior, which is one of the important influencing factors of their own problem behavior [13]. Their attentional control [14], cognitive flexibility [15], and executive function [16] are in the critical period of rapid development and are more susceptible to the influence of parental behavior [17]. Therefore, based on the support provided by the above theories, this study intends to explore whether the technoference caused by smartphone use in the interactions between couples can affect children’s smartphone dependence through factors such as marital relationship and parenting. This study aims to provide empirical research evidence for the prevention and intervention of child smartphone dependence from the aspect of parental behavior to reduce the negative impact of smartphone dependence on child growth and development.

## 2. Research Model and Hypotheses

### 2.1. Technoference in Conjugal Interactions and Child Smartphone Dependence

The frequent use of smartphones and other technologies in the family has greatly changed the way family members interact with each other, and technoference also occurs frequently in daily life scenes, such as face-to-face communication, eating, or leisure time [18]. Previous studies on technoference mainly focused on two kinds of situations: the technoference in the couple’s interactions and the technoference in parent–child interactions. However, most previous studies focused on the relationship between technoference in parent–child interactions and child smartphone dependence. For example, researchers have found that technoference in parent–child interactions can directly affect child smartphone dependence [19,20]. Although previous studies have ignored the direct or indirect influence of conjugal technoference on child or adolescent smartphone dependence, one study suggested that adolescents who perceive parental phubbing are more likely to engage in smartphone dependence [21].

In addition to increasing the externalizing problem behaviors among children and adolescents, technoference in conjugal interactions can also affect family ecology. Indeed, studies have shown that technoference in conjugal interactions has a great impact on family ecology. On the one hand, in the context of couples interacting, technoference can cause conflicts [22] and damage the marital relationship [16,23]. Contemporarily, conflicts in couples and the destruction of relationships will lead to the deterioration of the family atmosphere, which will create pressure for children. To escape or relieve this pressure, children may be addicted to smartphones. On the other hand, technoference in conjugal interactions can also reduce cooperative parenting behaviors between couples and the quality of raising children [24,25]. Similarly, negative parenting induced by technoference is often accompanied by more problem behaviors in children [26].

From a theoretical perspective, according to the concept of ecological development, the family is the microsystem with which children have direct contact. It is an interactive organic whole containing complex interrelated components and structures [27]. The spillover hypothesis shows that a family ecological subsystem of emotion and behavior would be expressed in another subsystem [28,29], such as when the emotion in the subsystem of husband and wife migrates to the parental subsystem, which shows that marital relationships are positively correlated with parenting behavior and ultimately affect the problem behaviors of adolescents. Intergenerational transmission exists not only in values, sociality, and emotion but also in behavioral patterns [30]. To sum up, this study focuses on the direct and indirect influence mechanism of marital technoference on child smartphone dependence. Therefore, Hypothesis 1 is proposed as follows:

**Hypothesis** **1.** *Technoference in conjugal interactions is positively related to child smartphone dependence*.

### 2.2. The Mediating Role of Marital Conflict

Marital conflict may play a mediating role in the relationship between technoference in conjugal interactions and child smartphone dependence. Marital conflict refers to the fact that the husband and wife show disagreement, attitude, opposition, and emotional dissatisfaction in the face of a certain problem or even violent behavior, including verbal quarrels and physical conflicts [31,32]. Previous studies have found that the occurrence of technoference in conjugal interactions can directly lead to marital conflict [33,34,35]. At the same time, technoference also causes couples to spend less time together and have lower relationship satisfaction [23,36] and causes the deterioration of the quality of the relationship between husband and wife [22]. In addition, studies have confirmed that marital satisfaction is highly correlated with marital conflict [37], and there is a corresponding relationship between the development trajectory of the two in the first ten years of marriage; that is, when marital satisfaction decreases, marital conflict will increase [38].

In addition, marital conflict may lead to smartphone dependence in children and adolescents. The emotional security hypothesis [39] posits that the emotional insecurity of individuals is manifested in all aspects of family life, and children who perceive conflict between their parents will produce negative emotions. If children are in such a negative mood for a long time, it will be harmful to their physical and mental health, leading to some problematic behaviors [40]. Marital conflict of parents is an important risk factor for internet addiction among adolescents [41]. Indeed, a study on senior high school students found that marital conflict can directly lead to adolescent smartphone dependence [42], as well as other addictive behaviors, such as game addiction [43] and internet addiction [41]. Therefore, Hypothesis 2 is proposed as follows:

**Hypothesis** **2** . *Marital conflict plays a mediating role in the effect of technoference in conjugal interactions on child smartphone dependence*.

### 2.3. The Mediating Role of Coparenting

Coparenting refers to the process of parenting in which parents adopt consistent parenting goals, concepts, attitudes, and methods [44,45]. On the one hand, more recent work has shown that technoference in conjugal interactions can affect coparenting quality. McDaniel and Coyne found that mothers who perceived greater levels of technoference in their coparenting reported worse coparenting quality [3]. Moreover, researchers have suggested that poor-quality coparenting leads to lower levels of positive fathering behavior with their children [46]. On the other hand, poor-quality coparenting is associated with problem behaviors in children and adolescents. The coparenting ecological model proposed by Feinberg in 2003 points out that there is a direct path from coparenting to child adaptation. Studies have also found that coparenting is indeed a major source of variation in the changes that parenting brings to children [47]. Previous researchers also found that the more active coparenting behaviors parents have, the fewer problem behaviors their children have [48]. Positive and supportive coparenting can reduce problem behaviors of children and adolescents, improve their adaptive emotional adjustment ability, and enhance their social skills [49]. On the contrary, high levels of damaging coparenting are associated with more explicit behavioral problems [50]. After controlling for marital quality, coparenting independently predicts both external and internal problem behavior in children [51]. Based on the fact that smartphone dependence is also a problem behavior, we can speculate that coparenting can directly influence children’s smartphone-dependence behavior. Therefore, Hypothesis 3 is proposed as follows:

**Hypothesis** **3.** *Coparenting mediates the relationship between technoference in conjugal interactions and child smartphone dependence*.

### 2.4. A Chain Mediation Model

The foregoing demonstrates that marital conflict or coparenting has a mediating effect on the relationship of technoference in conjugal interactions and child smartphone dependence. Then, how do marital conflict and coparenting work together? According to the theory of family systems, marital conflict and coparenting belong to different family subsystems, marital conflict is subordinate to the subsystem of husband and wife, and coparenting is subordinate to the subsystem of parents. The spillover hypothesis indicates that the emotions and behaviors generated by the couple subsystem spill over into the parent subsystem [48]. Studies have consistently found a positive association between conjugal relationship satisfaction and effective coparenting [9,52,53]. That is, the disharmonious relationship between parents in marriage tends to have a negative impact on couple–parent–child interactions by destroying their collaborative parenting behaviors, thus affecting the development of children [29]. Liu and Wu found a spillover effect between marital satisfaction, which reflects marital relationship and quality, and parental coparenting behaviors [54]. Kitzmann also found in his research that fathers showed lower democratic cooperative parenting after conflicts with their spouses [55]. That is, the quality of cooperative parenting after marital conflict was more negative, affecting parental parenting behavior toward their children. Therefore, Hypothesis 4 is proposed as follows:

**Hypothesis** **4.** *The association between technoference in conjugal interactions and child smartphone dependence can be mediated sequentially by marital conflict and coparenting*.

**Figure 1:**
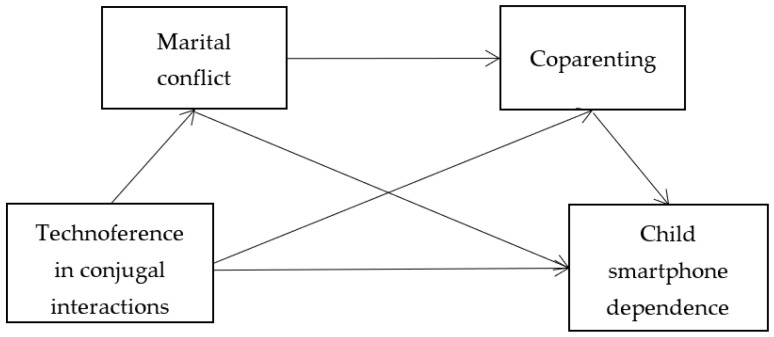
The hypothesized model.

Taken together, we mainly aim to test the research questions in the study (see Figure 1), that is, the influence mechanism of technoference in conjugal interactions on child smartphone dependence, as follows: technoference in conjugal interactions was not only directly correlated with child smartphone dependence, it can also indirectly affect children’s smartphone dependence through the mediating effect of marital conflict and coparenting.

## 3. Materials and Methods

### 3.1. Procedure and Participants

#### 3.1.1. Participants

We used a convenient sampling method to sample 545 primary schools in China’s economically developed coastal cities. The cluster random-sampling method was used to select several classes from each sample school, and all the students and parents of the selected classes were included in the sample on a voluntary basis. A total of 7796 students and their parents participated in the questionnaire survey. After clearing up the invalid questionnaires, 7436 valid questionnaires were left, and the questionnaire recovery rate was 95.38%. Before the formal analysis, the missing value mechanism of data cleaning was first carried out, and the analysis results showed that the Little S MCAR test results were significant, and the data were not completely randomly missing. However, the analysis of the degree of missing data found that the maximum missing value in all variables was only 0.3%, and the missing value of the data was 6.9%, all less than 10.0%. Therefore, the missing data were deleted according to the method of Liu [56]. Finally, our participants included 6923 fourth-grade children and their father or mother in 545 primary schools. The mean age of the children was 10.60 with a standard deviation of 0.70; among them, 55.0% were boys, and 45.0% were girls; 80.5% were single-child family, and 19.5% were multiple-child families. Of the parents, 35.3% were fathers and 64.7% were mothers. In addition, 31.0% of the families in this study were lower income level of socioeconomic status, 32.7% were middle income level of socioeconomic status, and 36.3% were higher income level of socioeconomic status.

#### 3.1.2. Data Collection

We gathered data from a regional educational quality assessment program in an economically developed coastal city of China. This program was similar to the Organization for Economic Cooperation and Development’s (OECD) Programme for International Student Assessment (PISA) and the International Association for the Evaluation of Educational Achievement’s (IEA) Trends in International Mathematics and Science Study (TIMSS). Such programs usually select a specific age or grade to represent a period of school stage. Similar to these programs, we selected fourth-grade students to represent primary school children. All sample schools received a letter of information that detailed the study’s purposes and procedures, and all the participants’ parents agreed that they could participate in this program. Students in the target classes were invited to participate anonymously in the survey in classrooms. Students completed the survey through the online questionnaire response system. The authenticity, independence, and integral nature of all answers and the confidentiality of the information collected were emphasized to all participants by well-trained psychology graduate students. It took the participants approximately 20 min to complete the questionnaire. Parents answered the online questionnaire at home by scanning the QR code issued by the sample school. It took about 10 min for parents and 20 min for students to answer the questionnaire. Each participant completed the measures independently in a self-administered format to safeguard confidentiality. All participation was voluntary, and the data were kept completely confidential.

### 3.2. Measures

#### 3.2.1. Technoference in the Conjugal Interactions

The questionnaire for measuring technoference in conjugal interactions was adapted from the Technology Interference in Life Examples Scale (TILES) and reported by parents [1]. First, considering that smartphones are most popular in families, studies have confirmed that smartphones are the devices with the highest interference frequency [1]. Therefore, to enhance the validity of the questionnaire survey, this study focused on smartphones with technology interference devices. Second, to make respondents accurately grasp the meaning of the options and better correspond to their own feelings, at the same time, the study emphasized the degree of technical interference rather than frequency; thus, we changed the original 8-point scoring method to a 4-point scoring method. Third, the newly revised questionnaire emphasized the negative impact of technological intervention, and the topic emphasized “playing with smartphone” to distinguish the necessary use of smartphones for work or life reasons. The new questionnaire included five questions (e.g., “My spouse has cut down on our conversations because she or he is always on her or his phone.”), which was answered by one of the parents. It was averaged on a 4-point Likert-style scale (1 = “strongly disagree” to 4 = “strongly agree”) with a higher score indicating more technology interference. Cronbach’s α for technoference in the conjugal interactions was 0.85.

#### 3.2.2. Marital Conflict Scale

The questionnaire for measuring marital conflict was adapted from the Marital Relationship Scale of the Chinese Children and Adolescents Psychological Development Characteristics Survey [31], and the evaluation of students in the original scale was changed to self-evaluation by parents [57]. The new questionnaire consisted of five items (e.g., “My spouse and I criticized and complained about each other at home.”), and the average score was calculated on a 5-point Likert-style scale (1 = “completely disagree” to 5 = “completely agree”). The higher the score, the more conflict the couple had. Cronbach’s α for marital conflict was 0.84.

#### 3.2.3. Coparenting Scale

The questionnaire for measuring coparenting used the Daily Coparenting Scale (D-COP) developed jointly by McDaniel, Teti, and Feinberg [53]. There are 10 items in total (e.g., “I share parental responsibilities with my spouse.”). The parents answered three reverse questions on a 7-point Likert-style scale (1 = “completely disagree” to 7 = “completely agree”), and the average score was calculated. The higher the score, the more harmonious the coparenting behavior was. Cronbach’s α for coparenting was 0.77. 

#### 3.2.4. Child Smartphone Dependence

A simplified version of the Mobile Phone Problem Use Scale (MPPUS-10) [58] was used to measure elementary students’ smartphone dependence, including 10 items (e.g., “When I am in a bad mood, I use my phone to make me feel better.”). The students answered the questions on a 5-point Likert-type scale (1 = “completely disagree” to 5 = “completely agree”), and the average score was calculated. The higher the score, the more dependent the individual was on mobile phones. Cronbach’s α for child smartphone dependence was 0.95.

#### 3.2.5. Covariates

In this study, we also collected information on parents’ gender (0 = “fathers”, 1 = “mothers”), children’s gender (0 = “boys”, 1= “girls”), socioeconomic status of the family (1 = “lower income level”, 2 = “middle income level”, 3 = “higher income level”) and the number of children (1 = “single-child”, 2= “multiple-child”). These variables were included as covariates given their associations with key study variables in prior studies [59,60,61].

### 3.3. Data Analysis

The Harman single-factor test was used to conduct a common method bias test for all the questions used in the model. It was found that there were five factors whose characteristic roots were greater than one. The variance explained by the first factor was 30.4%, less than the critical standard of 40.0%, indicating that there was no serious common method bias in this study [56,62,63]. In this study, SPSS 21.0 (IBM, Armonk, NY, USA) was used to conduct basic descriptive correlation analysis, Mplus 7.1 (Linda K. Muthén and Bengt O. Muthén, Los Angeles, CA, USA) was used to test mediating effects, and we randomly selected 5000 bootstrap examples from the data to estimate the 95% bias-corrected confidence intervals of the direct and indirect effects to examine whether there were statistically significant direct or indirect effects. If the confidence intervals did not include 0, the effect was considered significant [64]. 

## 4. Results

### 4.1. Preliminary Analyses

The means, SD, and Pearson correlations for the study variables are reported in Table 1. As expected, technoference in conjugal interactions was positively correlated with marital conflict and child smartphone dependence and negatively correlated with coparenting. Marital conflict was positively correlated with child smartphone dependence and negatively correlated with coparenting. Coparenting was negatively correlated with child smartphone dependence.

### 4.2. Testing the Mediating Effects of Marital Conflict and Coparenting

**Figure 2:**
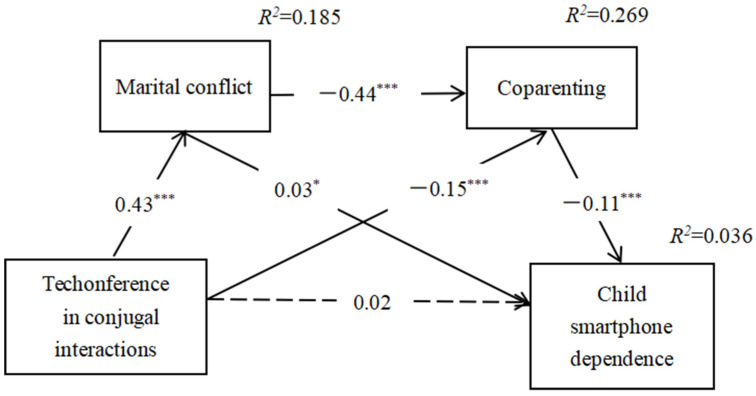
The chain mediation model. Note: the thin dotted line is not significant, and the solid black line is significant; covariates are not shown in the model diagram. * *p* < 0.05, *** *p* < 0.001.

Before testing the mediating model, we first conducted a simple linear regression between the technoference in the conjugal relationship and child smartphone dependence, and the results showed that the technoference in the conjugal relationship can significantly positively predict child smartphone dependence (*β* = 0.12, *p* < 0.001, *R*^2^ = 0.024). Then, marital conflict and coparenting were included as mediators in the association between technoference in the conjugal relationship and child smartphone dependence (see Figure 2), while controlling for the parents’ gender, children’s gender, the number of children, and socioeconomic status of the family. The fitting index of the chain mediation model was acceptable: *χ*^2^*/df* = 20.32(*χ*^2^ = 142.20, *df* = 7), CFI = 0.97, TLI = 0.93, RMSEA (90% CI) = 0.05 (0.045~0.061), and SRMR = 0.03 [65]. From Figure 2, technoference in the conjugal relationship positively predicted marital conflict (*β* = 0.43, *p* < 0.001) and negatively predicted coparenting (*β* = −0.15, *p* < 0.001). Additionally, marital conflict negatively predicted coparenting (*β* = −0.44, *p* < 0.001) and positively predicted (*β* = 0.03, *p* < 0.05) child smartphone dependence. Furthermore, the direct relationship between technoference in the conjugal relationship and child smartphone dependence was not significant (*β* = 0.02, *p* = 0.16), which indicated that marital conflict and coparenting completely mediated the relation between technoference in the conjugal relationship and child smartphone dependence.

We conducted a bias-corrected bootstrap test to understand whether the indirect paths were statistically significant. As Table 2 shows, after controlling for the number of children and socioeconomic status of the family, the three indirect paths were significant because 0 did not fall within the 95% confidence interval for the three indirect paths [66]. Whereas indirect path 1 (technoference in the conjugal relationship marital conflict child smartphone dependence) was 21.1%, indirect path 2 (technoference in the conjugal relationship coparenting child smartphone dependence) was 22.5%, and indirect path 3 (technoference in the conjugal relationship marital conflict coparenting child smartphone dependence) was 28.2%. The mediating effect accounted for 71.8% of the total effect.

## 5. Discussion

While considering the influence of technoference in conjugal interactions on child development and the spillover effect between different family subsystems, this study investigated the link between technoference in couples’ interactions and child smartphone dependence, as well as the possible mediating mechanisms underlying this relationship. The obtained findings not only contribute to the understanding of the relationship between technoference in conjugal interactions and child smartphone dependence but also provide a new perspective for the prevention and intervention of child smartphone dependence.

### 5.1. Technoference in Conjugal Interactions and Child Smartphone Dependence

This study explores the relationship between technoference in conjugal interactions and child smartphone dependence. The results show that there is a significant positive correlation between technoference in conjugal interactions and child smartphone dependence, and it can indirectly affect child smartphone dependence through the complete mediation mechanism of marital conflict and coparenting, which verifies our research hypothesis. This is consistent with previous research results of problematic parental smartphone use. For example, parents’ internet problem use behavior is also highly correlated with adolescents’ internet problem use behavior [67]. In addition, Hwang and Jeong also studied the influence of parents’ own smartphone use behavior on adolescents and found that parental smartphone dependence can positively influence adolescents’ smartphone dependence [68]. This also supports the family system theory and spillover effect, that is, behaviors and emotions between different subsystems of the family can be transmitted and influenced by each other.

### 5.2. The Mediating Role of Marital Conflict

Consistent with our hypothesis, we found that marital conflict mediated the relationship between technoference in conjugal interactions and child smartphone dependence. In other words, the technoference in conjugal interactions will increase the conflict between the couples, thus leading to child smartphone dependence. Therefore, marital conflict is not only the result of technoference in conjugal interactions but also the inducement of child smartphone dependence. This result is consistent with previous research results, that is, technoference increases conflict in interpersonal interactions and is one of the root causes of interpersonal conflict [1]. In addition, the results support the theory of social exchange. Specifically, the occurrence of technoference during couples’ interactions distracts and diverts spouses’ attention, which in turn reduces the time for effective interaction and greatly increases the cost of interaction between couples, leading to conflicts between couples [69].

In the second part of the mediation process, marital conflict increases child smartphone-dependence behavior. Previous research supports this hypothesis [42]. There are two possible reasons for this result. First, marital conflict is a negative experience for children in the family, and the negative emotions generated by such experiences can lead to addictive behaviors [70]. Second, parental care and warmth are important resources for child growth. A strong perception of verbal or physical conflict between children’s parents poses a threat to their inner security and means fewer support resources [71]. In this case, children will use smartphones more to compensate for the loss of parental support, leading to addictive behaviors.

### 5.3. The Mediating Role of Coparenting

Consistent with our hypothesis, coparenting was found to be another important mediator. Specifically, in the first half of the mediation process, the technoference in conjugal interactions leads to the reduction of coparenting behaviors. Coparenting is an act of cooperation and support between couples. When technoference appears in the interaction between couples, it will reduce the effective communication between couples, which will lead to the destruction of cooperative parenting behavior. This is also consistent with previous studies [3]. In the latter part of the mediation process, coparenting negatively affects child smartphone dependence. This is consistent with the compensation theory [8]. In other words, when parental coparenting behavior was disrupted, the parents showed more conflict in front of their children, which in turn triggered negative emotions in their children. At this point, the rich and open online world provides a platform for children to relieve negative emotions and compensate for their unmet psychological needs in the family. In addition, previous studies have found the same result [72].

### 5.4. The Chain Mediation Model

Finally, findings regarding the chain-mediating role of marital conflict and coparenting between technoference in conjugal interactions and child smartphone dependence means that parents who have a high level of marital conflict may have a lower level of coparenting. Thus, the H4 was verified. Our finding was consistent with previous studies in which researchers found a positive correlation between effective parenting behavior and relationship satisfaction [52]. In other words, marital conflict can destroy cooperative parenting relationships [50] and is the most important influencing factor of coparenting behavior [73]. This may be because when a parental relationship is in conflict, the parent’s focus shifts to his or her own relationship with the spouse, and the parent shifts from nurturing behavior to dealing with conflict. Research has also shown that conflict does reduce the quality of parenting for couples [17]. These findings suggest that in the context of couples interacting, the more technoference there is, the more likely it is to lead to conflicts in couples, which in turn leads to the disruption of coparenting behaviors and ultimately increases the risk of elementary students’ smartphone dependence.

Integrating the whole model, we find that the direct effect of technoference in conjugal interactions on child smartphone dependence is not significant, and the influence of marital conflict on smartphone dependence also is relatively small. In other words, the technoference in conjugal interactions indirectly affects child smartphone dependence through the mediating effect of coparenting and the chain-mediating effect from marital conflict to coparenting, and coparenting has a greater effect. This result not only proves the spillover effect but also verifies the coparenting model. The ecological model of coparenting proposed by Feinberg argues that coparenting is an intermediary process between the couple’s relationship and child adaptation [45]. In other words, no matter the technoference in conjugal interactions or marital conflict, as an important relational variable of the interaction between husband and wife, it is a remote influencing factor for children that generally plays a role in children’s behavior through coparenting. This also is consistent with previous findings that coparenting is an important mediator between the remote family environment and child development [51]. On the other hand, this study found that parent-reported marital conflict has a spillover effect on child smartphone dependence, but the effect is small. This may be because marital conflict in this study was reported by parents, while previous studies about the influence of the perceived conflicts between parents reported by children on smartphone dependence found that the perceived marital conflict by children is one of the important influencing factors of smartphone dependence [74] whose effect is much greater than the conflict effect between couples reported by parents. This can be interpreted as follows: when conflicts occur between husband and wife, the parents withdraw from parenting and deal with the problems between husband and wife, reducing their attention to their children and causing the children to engage in problem behaviors [75]. Therefore, the children’s perceptions of marital conflict are far more damaging than the objective conflict reported by parents.

In summary, based on the spillover hypothesis of the family system theory, this study comprehensively reveals the mediating mechanism of marital conflict and coparenting in the relationship between marital situational technological interference and child smartphone dependence. Most importantly, the results of this study provide the new perspective of parental factors intervening in child smartphone dependence. That is, parents need to start with themselves not only to reduce the frequent occurrence of technoference in the conjugal interactions but also to strengthen the effective communication and interactions between husband and wife with scientific and effective cooperative parenting behavior and then reduce the possibility of child smartphone dependence. After all, the family is a whole, and the emotional and behavioral influence generated between husband and wife will spill over into the parent–child relationship, thus affecting the development of children. Therefore, families and parents should pay attention to the harmony of the environment and relationship, effectively manage technoference, and maximize the support and protection of families and parents to reduce the occurrence of child smartphone dependence and other problem behaviors.

### 5.5. Limitations, Recommendations and Contribution

Several limitations need to be considered when interpreting the findings, starting with our cross-sectional data limit causal inferences. However, when the mediation models are based on theory and are partially supported by previous empirical research, cross-section mediation can provide valuable information about the relationship of variables. Future research should use longitudinal designs to test this multiple mediation model. Second, the present study used the participants’ self-reports to collect data. In the future, more diverse data acquisition methods can be used to quantify the technical interference. As others have done (e.g., [1,3]), we call for future researchers to examine these causal relationships more directly with longitudinal and daily diary studies. Additionally, our study explored the mechanism of technoference in conjugal interactions on child smartphone dependence from the perspective of family relationship and parenting, but there may be other personality and relationship variables that are yet unexplored that may be causal, moderating or mediating factors in the relationship between technoference in conjugal relationships and child smartphone dependence. According to the Interaction of Person-Affect-Cognition-Execution (I-PACE) model of specific internet-use disorders [76], there may be some mediation or mediation mechanism that explained how technoference in conjugal interactions might accelerate child smartphone dependence through personal emotional and cognitive factors, such as depression [22], couples’ distraction with technology, emotional stability [77], and so on.

Despite these limitations, our study still has theoretical and practical value. From a theoretical point of view, our study identifies the mechanism of technoference in conjugal interactions on child smartphone dependence. On the one hand, the results confirm the family system theory and the spillover hypothesis, that is, the family is a whole, and the technoference caused by one parent’s smartphone-use behavior in conjugal interactions will lead to an increase in child smartphone dependence by affecting conjugal interactions and parenting behavior. On the other hand, the effects of technoference in conjugal interactions on marital conflict and coparenting can be described using the media displacement theory, which proposes that people do not have unlimited time and attention and participating in a different communication activity can prevent an individual from participating with other people. Based on the position of this theory, spending time on devices, such as cell phones may reduce the number of meaningful interactions individuals can have with their spouses, which could reduce couples’ relationship quality and undermine coparenting [78]. Our results provide more evidence for the development of the family system theory, spillover hypothesis, and media displacement theory and have certain theoretical contributions.

From a practical point of view, this study provides an empirical framework for future intervention practices by testing the chain-mediating effects of marital conflict and coparenting. First, when the use of smartphones in the family becomes normal, most parents think that the use of smartphones will not affect their children as long as they do not use it when they get along with their children. However, our research results show that the interference caused by smartphone use between husband and wife will also affect child smartphone dependence. In the second place, parents should use smartphones reasonably, strengthen emotional communication and positive interactions between husband and wife, and create a good relationship between husband and wife and the family atmosphere. In other words, only if the parents can properly use the electronic technology to communicate and the relationship is harmonious, can the children have less smartphone-dependence behavior and perform better in the future growth and development. Last but not least, this study found that the mediating effect of marital conflict was small, while the mediating effect of coparenting and chain mediation was large; thus, parents should pay attention to the interactions between each other on the comprehensive impact of family ecology. Additionally, more attention should be paid to the interactions and cooperation between parents, especially in the aspect of parenting. Only more cooperative parenting behaviors can reduce children’s smartphone dependence.

## 6. Conclusions

We identified the association between technoference in conjugal interactions and child smartphone dependence and the mediating roles of marital conflict and coparenting. First, technoference in conjugal interactions can increase child smartphone dependence by inducing conjugal conflict, and conjugal conflict played a mediating role. Secondly, technoference in conjugal interactions can increase child smartphone dependence by disrupting coparenting, and coparenting played a mediating role. In addition, marital conflict and coparenting played chain-mediating roles in technoference in conjugal interactions on the effect of child smartphone dependence. Our findings provide promising directions for interventions to reduce smartphone dependence among children. Interventions should focus on reducing inappropriate use of electronic technology in couples’ interactions, improving the conjugal relationship, and enhancing coparenting behaviors to achieve optimal intervention outcomes.

## Figures and Tables

**Table 1:** Descriptive statistics and intercorrelations between variables.

	Mean	SD	1	2	3	4
1 Technoference in conjugal interactions	1.98	1.05	-			
2 Marital conflict	2.10	0.83	0.11 **	-		
3 Coparenting	5.51	0.95	−0.14 **	−0.50 **	-	
4 Child smartphone dependence	2.28	1.28	0.71 **	0.10 **	−0.14 **	-

Note: ** *p* < 0.01, *n* = 6923; SD indicates “standard deviation”; 1 indicates technoference in conjugal interactions; 2 indicates marital conflict; 3 indicates coparenting; 4 indicates child smartphone dependence.

**Table 2:** Bias-corrected bootstrap test on the mediating effects.

Path	Standardized *β*	95% CI	
		BootLLCI	BootULCI
X → M1 → Y	0.015	0.002	0.027
X → M2 → Y	0.016	0.011	0.021
X → M1 → M2 → Y	0.020	0.015	0.026
Indirect effects	0.051	0.039	0.063
Total effect	0.071	-	-

Note: X= Technoference in conjugal interactions, M1 = marital conflict, M2 = coparenting, Y = child smartphone dependence.

## Data Availability

The datasets analyzed during the current study are not yet publicly available but are available from the corresponding authors upon reasonable request.
